# Genome wide analysis of DNA methylation and nucleosome positioning

**DOI:** 10.1186/1756-8935-6-S1-O37

**Published:** 2013-03-18

**Authors:** Peter A Jones

**Affiliations:** 1 Department of Urology and Biochemistry & Molecular Biology, USC Norris Comprehensive Cancer Center, Keck School of Medicine of USC, Los Angeles, CA, USA

## 

DNA methylation and nucleosome positioning work together to generate chromatin structures that regulate gene expression. Nucleosomes are typically mapped using nuclease digestion but we have developed a method (NOMe-Seq) that uses a GpC methyltransferase (M.CviP1) and next generation sequencing to generate high resolution footprints of nucleosome positioning genome wide using less than 1 million cells while retaining endogenous DNA methylation information from the same strand. We have developed a novel bioinformatics pipeline that shows a striking anti-correlation between nucleosome occupancy and DNA methylation at CTCF regions that is not present at promoters. The extent of nucleosome depletion of promoters is directly correlated with expression level and can accommodate multiple nucleosomes. Genome wide evidence also shows that expressed non-CpG island promoters are nucleosome depleted. Because the method obtains DNA methylation and nucleosome positioning information from the same DNA molecule we can also use this method to track the relationship between nucleosome occupancy and DNA methylation during differentiation and mitosis. These experiments have shown that nucleosome occupancy precedes DNA methylation in the OCT4 promoter which is consistent with the idea that nucleosomal DNA is the preferred substrate for *de novo* DNA methylation. We have also used this approach to show that nucleosomes shift during mitosis to cover the transcription start sites of genes. This may be a mechanism to bookmark genes for expression in the following G1 phase of the cell cycle.

